# Long Non-coding RNA TMEM220-AS1 Suppressed Hepatocellular Carcinoma by Regulating the miR-484/MAGI1 Axis as a Competing Endogenous RNA

**DOI:** 10.3389/fcell.2021.681529

**Published:** 2021-08-05

**Authors:** Cong Cao, Jun Li, Guangzhi Li, Gaoyu Hu, Zhihua Deng, Bing Huang, Jing Yang, Jiequn Li, Song Cao

**Affiliations:** ^1^Department of General Practice, The Affiliated Hospital of Youjiang Medical University for Nationalities, Baise, China; ^2^Department of Gastroenterology, The Affiliated Hospital of Youjiang Medical University for Nationalities, Baise, China; ^3^Department of Liver Transplantation, Second Xiangya Hospital, Central South University, Changsha, China; ^4^Transplant Medical Research Center, The Second Affiliated Hospital of Guangxi Medical University, Nanning, China; ^5^Department of Liver Transplantation, The Second Affiliated Hospital of Guangxi Medical University, Nanning, China

**Keywords:** hepatocellular carcinoma, long non-coding RNA, TMEM220-AS1, cell invasion, epithelial-mesenchymal transition

## Abstract

Long non-coding RNAs (lncRNAs) have a considerable regulatory influence on multiple biological processes. Nevertheless, the role of TMEM220-AS1 in hepatocellular carcinoma (HCC) remains unclear. We used The Cancer Genome Atlas (TCGA) database to analyze the differentially expressed lncRNAs. qRT-PCR was used to verify the results for a large population. The *in vitro* effects of TMEM220-AS1 on HCC cells were determined using Cell Counting Kit-8 (CCK-8), 5-ethynyl-2’-deoxyuridine (EdU), flow cytometry, and Transwell assays in HCC cells. We used qRT-PCR and western blotting to identify the epithelial-mesenchymal transition (EMT). Moreover, we performed bioinformatics analysis, western blotting, dual luciferase reporter gene assay, RNA pull-down, and RNA binding protein immunoprecipitation (RIP) to investigate the underlying molecular mechanisms of TMEM220-AS1 function. Finally, the function of TMEM220-AS1 was verified *in vivo*. The results showed that TMEM220-AS1 was expressed at considerably low levels in HCC. It was demonstrated that malignant phenotypes and EMT of HCC cells were promoted by the knock down of TMEM220-AS1 both *in vivo* and *in vitro*. TMEM220-AS1, which was detected primarily in the cytoplasm, functioned as an miRNA sponge to bind miR-484 and promote the level of membrane-associated guanylate kinase, WW, and PDZ domain containing 1 (*MAGI1*), thereby curbing the malignant phenotypes of HCC cells. In conclusion, low levels of TMEM220-AS1 promote proliferation and metastasis through the miR-484/*MAGI1* axis in HCC.

## Introduction

As the sixth most frequently occurring cancer worldwide, liver cancer is the third leading cause of cancer-related deaths, globally (Nakagawa et al., 2019; Anwanwan et al., 2020; Rahmani et al., 2020). Among all primary liver cancers, hepatocellular carcinoma (HCC) is the most frequent, accounting for 80–90% of all cases (Ringelhan et al., 2017). HCC, which is one of the most aggressive and resistant cancers, has a poor prognosis (Gailhouste et al., 2018). In the United States and many other countries, the morbidity of HCC has doubled over the past two decades. Annually, the number of patients diagnosed with HCC is almost 800,000 worldwide, with approximately 750,000 causalities (Ryerson et al., 2016; Momin et al., 2017). Chronic hepatitis B and C viral infections are the most common risk factors and are responsible for approximately 75% of HCCs, leading to a twenty-fold increase in the development of HCCs (Ryerson et al., 2016). Other major risk factors include non-alcoholic fatty liver disease (NAFLD), aflatoxin B1 (AFB1) exposure obesity, and chronic alcohol consumption (Kim et al., 2014). However, the molecular mechanisms involved in the pathogenesis of HCC are still under intense investigation.

Recently, increasing evidence has identified lncRNAs as vital regulators in numerous cancers, including HCC (Chi et al., 2020; Zhou et al., 2020). Abnormal lncRNA expression exerts a considerable influence on cancer progression and carcinogenesis through several mechanisms (Lian et al., 2018; Tichon et al., 2018). For instance, LINC00346 modulates the CDK1/CCNB1 axis, consequently regulating the development of HCC and serving as a competing endogenous RNA (Jin et al., 2020). In HCC, LINC00160 mediates drug resistance and autophagy through the microRNA-132/PIK3R3 axis (Zhang et al., 2020). By modifying the genomic methylation profiles, LINC00662 can promote the progression of HCC progression (Guo et al., 2020).

We used TCGA database to analyze the differentially expressed lncRNAs and found that TMEM220-AS1 was poorly expressed in HCC; however, it is unclear whether TMEM220-AS1 is correlated with the development of HCC. To determine this, we assessed the function of TMEM220-AS1 in HCC by performing a large sample validation in a population, followed by a series of cell function tests, dual luciferase reporter gene assay, bioinformatics analysis, western blotting, and RNA binding protein immunoprecipitation (RIP) to explore the underlying molecular mechanisms of TMEM220-AS1 function. We verified that TMEM220-AS1 is a novel tumor suppressor that regulates HCC through the miR-484/*MAGI1* axis.

## Materials and Methods

### Collection of Clinical Samples

From 2016 to 2018, 50 paired fresh liver tumor and adjacent normal tissues were harvested at The Affiliated Hospital of Youjiang Medical University for Nationalities. We snap-freezed these tissues at −80°C. All included subjects offered an informed consent and the research got approval from the Institutional Review Board of The Affiliated Hospital of Youjiang Medical University for Nationalities.

### Cell Culture

HB611, HHCC, H-97, HuH-7, Li-7, and LO2 cell lines were acquired from the American Type Culture Collection (ATCC, Manassas, VA, United States) and the Cell Bank of the Chinese Academy of Sciences (Shanghai, China). Human immortalized liver LO2 cells were cultured in Dulbecco’s modified Eagle’s medium (DMEM; Gibco, United States). We cultured HCC cells in DMEM with high glucose concentration (25 mM), 1% penicillin-streptomycin, and 10% fetal bovine serum (FBS), and maintained them in a 5% CO_2_ humidified incubator.

### Cell Transfection

We purchased plasmid vector PLKO.1-puro from BioVector NTCC Inc., Guangzhou, China. Through chemical synthesis, we designed the related TMEM220-AS1 and *MAGI1* short hairpin RNA (shRNA) sequences (Table 1) and the negative control. These synthesis-related sequences were inserted into the PLKO.1-puro vector. We purchased microRNA mimics and their inhibitors from RIBOBIO, Guangzhou, China. Cells were cultured for 24 h before transfection. We then transiently transfected the cells with the corresponding vector, using Lipofectamine 3000 Transfection Reagent (Invitrogen, Carlsbad, CA, United States) as per the manufacturer’s instructions. We harvested cells that were transfected with the corresponding vector and performed quantitative real-time polymerase chain reaction (qRT-PCR) after 48 h. Each experiment was performed in triplicate.

**TABLE 1:** qRT-PCR or shRNAs related sequences.

Name		Sequence
	Forward	5′-AGCTTCCACTCTTGTCTCCC-3′
	Reverse	5′-TGAGCAGTGATGGAGCAGAA -3′
TMEM220-AS1		
	Forward	5′-ACACTCCAGCTGGGUAGCCCU CCCCUGACU-3′
	Reverse	5′-CTCAACTGGTGTCGTGGAGTCG GCAATTCAGTTGAGAGTCCGAG-3′
miR-484		
	Forward	5′-GAACAAGGACCTGCGACATTT -3′
	Reverse	5′-ACAGCATGGCGGTAAAGGTTA -3′
MAGI1		
	Forward	5′- GCTGGACCGAGAGAGTTTCC -3′
	Reverse	5′- CAAAATCCAAGCCCGTGGTG -3′
E-cadherin		
	Forward	5′- CGGGAGAAATTGCAGGAGGA -3′
	Reverse	5′-AAGGTCAAGACGTGCCAGAG-3′
Vimentin		
	Forward	5′-TCGGAAGCCTAACTACAGCGA-3′
	Reverse	5′-AGATGAGCATTGGCAGCGAG-3′
Snail		
	Forward	5′- GATCCCCTGTAATCCCAGCTACT CAGCTTCCTGTCACTGAGTAGCTGG GATTACATTTTTGGAAA-3′
	Reverse	5′- AGCTTTTCCAAAAATGTAATCCCA GCTACTCAGTGACAGGAAGCTGAGT AGCTGGGATTACAGGG-3′
TMEM220-AS1 shRNA #1		
	Forward	5′- GATCCCCTGGTGAAACCCCGTA TCTCCTTCCTGTCAGAGATACGGG GTTTCACCATTTTTGGAAA-3′
	Reverse	5′- AGCTTTTCCAAAAATGGTGAAACC CCGTATCTCTGACAGGAAGGAGATAC GGGGTTTCACCAGGG-3′
TMEM220-AS1 shRNA #2		

### RNA Isolation and qRT-PCR

Total RNA was extracted from cell samples using TRIzol reagent (Invitrogen). Referring to the manufacturer’s instructions, RNA was reverse transcribed using the PrimerScript RT-PCR kit (Takara). RNA levels were determined using qRT-PCR analysis using the TaqMan MicroRNA Assay Kit (Applied Biosystems). We measured the relative levels of the predicted targets in triplicate on an ABI 7500 real-time PCR machine (Applied Biosystems). U6 or β-actin was used as a reference gene to normalize the expression levels of miRNAs or mRNAs. The delta Ct method was used to calculate the relative expression. The primers used in this study are shown in Table 1.

### Cell Proliferation, Invasion, Cycle, and Apoptosis Detection

These methods are shown in Supplementary Methods.

### Western Blotting

Total cell lysates were prepared in 1 × sodium dodecyl sulfate buffer. Next, the proteins were separated by sodium dodecyl sulfate-polyacrylamide gel electrophoresis, and total proteins were transferred onto nitrocellulose membranes. Then, with 5% non-fat milk, the membrane was blocked and incubated with primary antibodies at 4°C overnight. After incubation with antibodies specific for β-actin (ab8227, Abcam, Hong Kong, China), MAGI1 (55048-1-AP, WUHAN SANYING, Wuhan, China), E-cadherin (ab227639), vimentin (ab92547), and snail (ab216347), the membrane was incubated with goat anti-rabbit secondary antibody (ab7090) and visualized via enhanced chemiluminescence. Each experiment was performed in triplicate.

### RNA Fluorescent *in situ* Hybridization (FISH)

The FISH assay was implemented using Ribo^TM^ Fluorescent *in situ* Hybridization Kit (Ribobio Company, China). The TMEM220-AS1 probe was labeled with FITC fluorescent dye, and the design and synthesis were implemented by Ribobio Company. RNA FISH was performed using a fluorescent *in situ* hybridization kit (RiboBio) following the manufacturer’s instructions. Fluorescence was detected using a confocal laser scanning microscope (Leica, Germany).

### RIP Assay

Following the product specifications, we adopted the EZ-magna RIP kit (Millipore, United States) to perform the RIP assay. HB611 and HuH-7 cells were collected and lysed in a full RIP lysis buffer. Cell extracts were incubated with RIP buffer containing magnetic beads conjugated to human AGO2 antibodies (ab32381, Abcam, Cambridge, United Kingdom); we used the IgG antibody (ab6702, Abcam) as control. Samples were incubated with protease K, and oscillated to digest the protein and isolate the immunoprecipitated RNA. Using a NanoDrop spectrophotometer, we measured the concentration of RNA and performed real-time PCR analysis using the purified RNA.

### Dual Luciferase Reporter Gene Assay

First, we manufactured TMEM220-AS1 Wt and *MAGI1* Wt. In brief, TMEM220-AS1 and *MAGI1* fragments containing miR-484 binding sites were amplified using PCR and cloned downstream of the luciferase reporter gene in the pmirGLO vector, which were named TMEM220-AS1 Wt and *MAGI1* Wt. Using the Quickchange XL Site-Directed Mutagenesis Kit (Stratagene), we generated TMEM220-AS1 Mut and *MAGI1* Mut (mutations within the binding sites). MiR-NC and miR-484 mimic were co-transfected with TMEM220-AS1 Wt or TMEM220-AS1 Mut and *MAGI1* Wt or *MAGI1* Mut, respectively, into HEK293T cells. Cells were harvested 48 h after transfection and the Dual-Luciferase Reporter Assay System (Promega, Madison, WI, United States) was used to perform the luciferase assay.

### Immunochemistry

To detect Ki-67 staining in tumor tissue samples, sections of 5 μm were cut. After dewaxing and hydration, the slides were rinsed in PBS, followed by boiling in 10 mM sodium citrate at pH 6. Then, the slides were incubated in 3% H_2_O_2_ for 25 min to remove horseradish peroxidase. The slides were blocked with 10% BSA after washing thrice with 1 × PBS, followed by incubation with primary anti-Ki-67 antibody (ab92742) at 4°C overnight. The slides were incubated with a secondary antibody labeled with HRP (rabbit) at room temperature for 45 min and with 3,3-diaminobenzidine tetrahydrochloride (DAB), and the immunoreactivity was visualized the next day. Finally, the slides were dehydrated and mounted with neutral gum.

### Tumor Xenograft Implantation in Nude Mice

Six-week-old nude mice were randomly divided into two groups (three mice per group), and cultured with continuous access to sterile food and water in pathogen-free sterile conditions. For transfections, cells at 60–80% confluence were infected with 1 × 10^6^ recombinant lentivirus-transducing units and 6 μg/mL Polybrene (Sigma). Stably transfected cells were selected using 2 μg/mL puromycin treatment for 2 weeks. Stably transfected cells were selected for subsequent assays via flow cytometry. Lentivirus used in this study was purchased from GenePharma (Shanghai, China). To establish the HCC xenograft model, we subcutaneously injected 5 × 10^6^ HB611 cells stably transfected with *MAGI1* overexpression vectors or TMEM220-AS1 overexpression vectors into nude mice. Tumor growth was monitored weekly and calculated as follows: volume = (length) × (width)^2^/2. The study was approved by the Ethics Committee of The Affiliated Hospital of Youjiang Medical University for Nationalities, and experiments were performed following the NIH guidelines on animal welfare.

### Lung Metastasis Assay

Briefly, 1 × 10^6^ HB611 cells in 30 μL of 30% Matrigel were injected intravenously through the tail vein of nude mice. After 6 weeks, the mice were sacrificed, and metastatic nodules in each lung were analyzed. All animal experiments were performed according to the protocols approved by the Animal Experimental Ethics Committee of The Affiliated Hospital of Youjiang Medical University for Nationalities.

### Statistical Analysis

For normally distributed data with equal variance, the difference was evaluated using a two-tailed Student’s *t*-tests (two-group comparisons) or ANOVA, followed by the Bonferroni’s *post hoc* test (multigroup comparisons). For non-normally distributed data or data with unequal variances, the difference was evaluated using a non-parametric Mann–Whitney *U*-test (two-group comparisons) or the Kruskal–Wallis test followed by the Bonferroni’s *post hoc* test (multigroup comparisons). *P* < 0.05 determined statistical significance. All tests were performed using SPSS (version 22.0, SPSS, Chicago, IL, United States).

## Results

### Low Level of TMEM220-AS1 in HCC Tissues and Cell Lines

**FIGURE 1:**
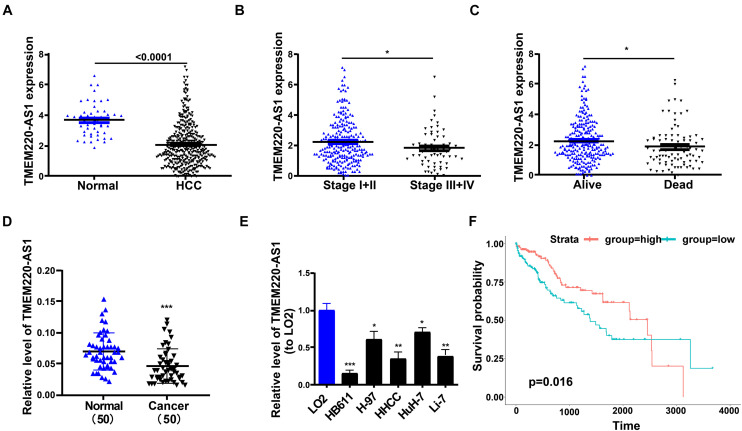
Low level of TMEM220-AS1 in HCC tissues and cell lines. **(A)** TME M220-AS1 expression in HCC samples and normal samples, from TCGA database. **(B)** TMEM220-AS1 expression in stage I + II and stage III + IV, from TCGA database. **(C)** TMEM220-AS1 expression in alive samples and dead samples, from TCGA database. **(D)** TMEM220-AS1 expression in HCC tissues and paired adjacent normal tissues was analyzed by qRT-PCR. **(E)** TMEM220-AS1 expression in HCC cell lines and LO2 cell line was analyzed by qRT-PCR. **(F)** Kaplan-Meier curves of overall survival (OS) from TCGA database. Data represent the mean ± SD; *P < 0.05, **P < 0.01, ***P < 0.001.

Through analysis of the TCGA database, we found that TMEM220-AS1 was remarkably lower in HCC tissues than that in normal tissues (Figure 1A). Second, the expression level of TMEM220-AS1 in periods III and IV was lower than that in periods I and II (Figure 1B). TMEM220-AS1 expression levels in the tissues that were dead were lower than those in the tissues that were alive (Figure 1C). We verified this result in 50 HCC tissues and adjacent non-tumorous tissues. As revealed by qRT-PCR assays, TMEM220-AS1 levels were remarkably lower in HCC tissues than those in paired adjacent normal liver tissues (Figure 1D). We detected the mRNA level of TMEM220-AS1 in six cell lines, including one normal cell line (LO2) and five HCC cell lines (HB611, HHCC, H-97, HuH-7, and Li-7). Similarly, TMEM220-AS1 was found to be expressed at low levels in HCC cell lines compared to those in LO2 cells (Figure 1E). Among the HCC cell lines, the expression level of TMEM220-AS1 was the highest in HuH-7 cells and the lowest in HB611 cells. Therefore, HuH-7 and HB611 cell lines were used as cell models in subsequent studies. Data from TCGA database showed that the overall survival rate of patients with low TMEM220-AS1 levels was lower than that of patients with high TMEM220-AS1 levels (Figure 1F).

### TMEM220-AS1 Inhibits Proliferation and Cell Cycle of HCC Cells, and Promotes Cell Apoptosis of HCC Cells

**FIGURE 2:**
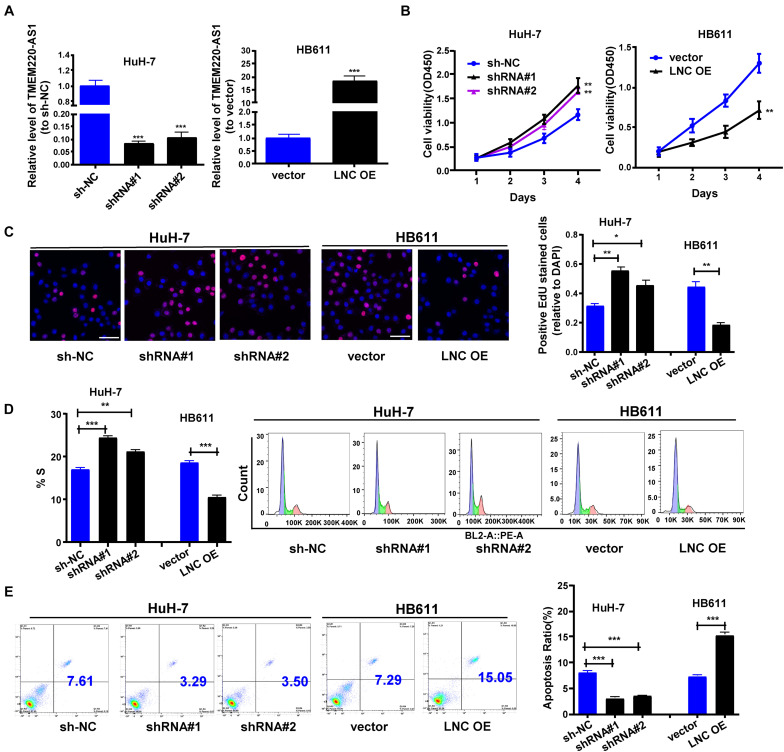
TMEM220-AS1 inhibits the proliferation and cell cycle of HCC cells, but promotes the cell apoptosis of HCC cells. **(A)** Transfection efficiency of sh-TMEM220-AS1#1 (shRNA#1), sh-TMEM220-AS1#2 (shRNA#2) and TMEM220-AS1-overexpressing vector (LNC OE). **(B)** Cell viability was analyzed by **(B)** CCK-8 assay and **(C)** EdU assay (bar = 50 μm). **(D)** Cell cycle. **(E)** Cell apoptosis. Data were presented as represent the mean ± SD of 3 independent experiments; *P < 0.05, ** *P* < 0.01, ***P < 0.001.

Two shRNAs targeting different sites of TMEM220-AS1 mRNA were used to knockdown TMEM220-AS1 in HuH-7 cells (Figure 2A). Using a TMEM220-AS1-overexpressing vector, we overexpressed TMEM220-AS1 in HB611 cells (Figure 2A). CCK-8 demonstrated that TMEM220-AS1 knockdown remarkably promoted proliferation in HuH-7 cells, and overexpression of TMEM220-AS1 remarkably suppressed proliferation in HB61 cells (Figure 2B). Similar promotional effects of TMEM220-AS1 on HCC proliferation were also demonstrated by EdU assays (Figure 2C). The proportion of cells in the S phase increased when transfected with the TMEM220-AS1 shRNA, while it was decreased by TMEM220-AS1 overexpression (Figure 2D). Additionally, TMEM220-AS1 elevated the apoptotic rate of HB611 cells, while TMEM220-AS1 knockdown remarkably suppressed the apoptosis of HuH-7 cells (Figure 2E).

### TMEM220-AS1 Inhibits Cell Invasion and EMT of HCC Cells

**FIGURE 3:**
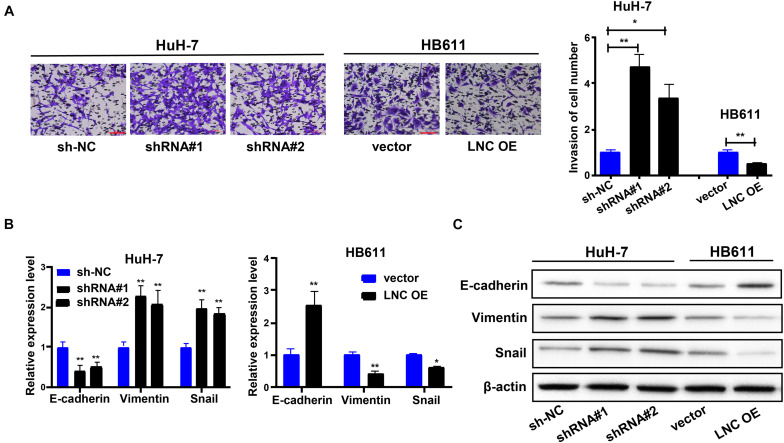
TMEM220-AS1 inhibits the cell invasion and EMT of HCC cells. **(A)** Cell invasion was determined using Transwell assay (bar = 100 μm). **(B,C)** The expression of EMT markers were determined using qRT-PCR and western blot assay. Data were presented as represent the mean ± SD of 3 independent experiments; *P < 0.05, **P < 0.01.

Next, we investigated whether TMEM220-AS1 regulates the invasion of HCC cells. Using the Transwell assay, the invasive ability of HCC cells was identified. Inhibited cell invasion was observed in HB611 cells transfected with the TMEM220-AS1-overexpressing vector. In contrast, TMEM220-AS1 knockdown increased cell invasion (Figure 3A). We also explored whether TMEM220-AS1 regulates the EMT of HCC cells. We used qRT-PCR and western blotting to observe the expression of EMT markers. E-cadherin expression was decreased while Snail and vimentin expression was increased by TMEM220-AS1 knockdown in HCC cells (Figures 3B,C).

### TMEM220-AS1 Interacted With miR-484 in a Direct Manner

**FIGURE 4:**
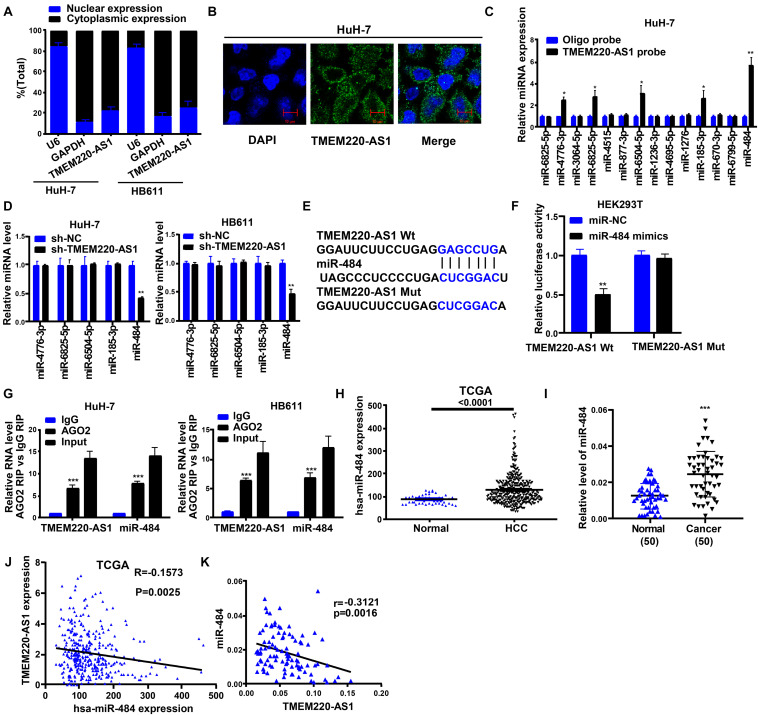
TMEM220-AS1 is targeted by miR-484. **(A,B)** Localization of TMEM220-AS1 by nucleocytoplasmic separation experiment and RNA-FISH in HCC cells (bar = 10 μm). **(C)** The relative expression of candidate miRNAs which could potentially bind to TMEM220-AS1 were quantified by qRT-PCR after the biotinylated- TMEM220-AS1 pull-down assays in HuH-7 cells. **(D)** The levels of miRNAs after TMEM220-AS1 cutdown were tested by qRT-PCR. **(E)** Putative miR-484 binding sequence and mutation sequence of TMEM220-AS1 mRNA were as shown. **(F)** Dual luciferase reporter assays were used to confirm the direct target between TMEM220-AS1 and miR-484. **(G)** RIP assay was used to detect whether miR-484 could bind with TMEM220-AS1. **(H)** miR-484 expression in HCC samples and normal samples, from TCGA database. **(I)** The correlation analysis between miR-484 expression and TMEM220-AS1 expression in HCC samples and normal samples, from TCGA database. **(J)** miR-484 expression in HCC tumor tissues and adjacent non-tumorous tissues. **(K)** The correlation analysis between miR-484 expression and TMEM220-AS1 expression in HCC tumor tissues and adjacent non-tumorous tissues, from our dataset. Data were presented as represent the mean ± SD; *P < 0.05, **P < 0.01, ***P < 0.001.

The biological effects and potential molecular roles of lncRNAs are closely associated with their subcellular localization (Wen et al., 2018). We performed a nucleocytoplasmic separation experiment to determine the subcellular distribution of TMEM220-AS1. It was found that most TMEM220-AS1 was concentrated in the cytoplasm, with a minority in the nucleus (Figure 4A). Moreover, this was confirmed by the RNA-FISH assay (Figure 4B). To uncover the underlying mechanisms of TMEM220-AS1 function, we searched for potential targets using the LncBase Experimental v.2. Fourteen miRNAs (hsa-miR-6825-5p, hsa-miR-4776-3p, hsa-miR-3064-5p, hsa-miR-6825-5p, hsa-miR-4515, hsa-miR-877-3p, hsa-miR-6504-5p, hsa-miR-1236-3p, hsa-miR-4695-5p, hsa-miR-1276, hsa-miR-185-3p, hsa-miR-670-3p, hsa-miR-6799-5p, and hsa-miR-484) with a score greater than 0.85 were selected as potential research objects. The results of the RNA pull-down assay with biotin-labeled TMEM220-AS1 in HuH-7 cells showed that hsa-miR-4776-3p, hsa-miR-6825-5p, hsa-miR-6504-5p, hsa-miR-185-3p, and hsa-miR-484 could be pulled down by TMEM220-AS1 (Figure 4C). Then, we silenced the expression of TMEM220-AS1 in HuH-7 and HB611 cells; only miR-484 was remarkably upregulated (Figure 4D). Therefore, we chosen miR-484 as the study subject. To further identify whether miR-484 could interact with TMEM220-AS1 directly, we conducted dual luciferase reporter and RIP assays. The binding sites of wild-type (TMEM220-AS1 Wt) and mutant-type (TMEM220-AS1 Mut) are shown in Figure 4E. Dual luciferase reporter assays in HEK293T cells demonstrated that luciferase activity was remarkably reduced by co-transfection with TMEM220-AS1 Wt and miR-484 mimics (Figure 4F). Using the RIP assay, we further validated the interaction between miR-484 and TMEM220-AS1. We found that both TMEM220-AS1 and miR-484 were enriched in AGO2-containing miRNA ribonucleoprotein complexes (Figure 4G). Consistently, both TCGA database and our dataset showed that miR-484 expression in HCC tumor samples was higher than that in negative control samples (Figures 4H,I). Moreover, TMEM220-AS1 expression levels were negatively correlated with miR-484 expression in HCC samples, both in the TCGA database and our dataset (Figures 4J,K). Taken together, the above results proved that TMEM220-AS1 was targeted by miR-484.

### TMEM220-AS1 Regulates the miR-484 Target Gene, MAGI1

**FIGURE 5:**
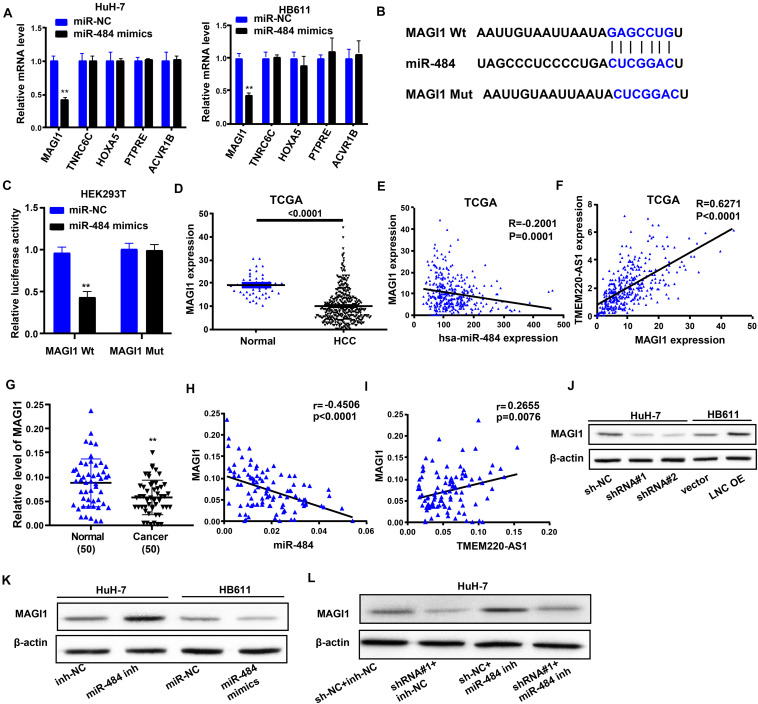
TMEM220-AS1 regulates the miR-484 target gene, *MAGI1.* **(A)** The levels of mRNAs after miR-484 cutdown were tested by qRT-PCR. **(B)** Putative miR-484 binding sequence and mutation sequence of *MAGI1* mRNA were as shown. **(C)** Dual luciferase reporter assays were used to confirm the direct target between *MAGI1* and miR-484. **(D)** *MAGI1* gene expression in HCC samples and normal samples, from TCGA database. **(E)** The correlation analysis between miR-484 expression and *MAGI1* expression in HCC samples and normal samples, from TCGA database. **(F)** The correlation analysis between TMEM220-AS1 expression and *MAGI1* expression in HCC samples and normal samples, from TCGA database. **(G)** *MAGI1* gene expression in HCC tissues and paired adjacent normal tissues, from our dataset. **(H)** The correlation analysis between miR-484 expression and *MAGI1* expression in HCC tissues and paired adjacent normal tissues, from our dataset. **(I)** The correlation analysis between TMEM220-AS1 expression and *MAGI1* expression in HCC tissues and paired adjacent normal tissues, from our dataset. **(J–L)** The level of MAGI1 was detected by western blot assay. Data were presented as represent the mean ± SD; **P < 0.01.

Target genes of miR-484 were screened out through MIRDB, and the top five mRNAs (*MAGI1*, *TNRC6C*, *HOXA5*, *PTPRE*, and *ACVR1B*) according to their scores were selected as potential research subjects. Only *MAGI1* expression was inhibited by miR-484 overexpression in HCC cells (Figure 5A). In addition, studies have shown that *MAGI1* inhibits cancer cell migration and invasion in HCC (Zhang and Wang, 2011; Zhang et al., 2012). Therefore, we chose *MAGI1* as the study object. We showed the binding sites of wild-type (*MAGI1* Wt) and mutant-type (*MAGI1* Mut) (Figure 5B). Dual luciferase reporter assays demonstrated that luciferase activity was remarkably reduced by *MAGI1* Wt and miR-484 mimic co-transfection (Figure 5C). Both the TCGA database and our dataset showed that *MAGI1* gene expression in HCC samples was lower than that in negative control samples (Figures 5D,G). Moreover, *MAGI1* expression levels were negatively correlated with miR-484 expression in HCC samples (Figures 5E,H), but it was positively correlated with TMEM220-AS1 expression in HCC samples, according to the TCGA database and our dataset (Figures 5F,I). Altogether, *MAGI1* was indicated to be a target gene of miR-484.

Next, we used western blotting to investigate whether TMEM220-AS1 can modulate the expression of MAGI1 in HCC cells via miR-484. The results showed that MAGI1 expression levels were inhibited by sh-TMEM220-AS1 and miR-484 mimics (Figures 5J,K). MAGI1 expression was promoted by TMEM220-AS1 overexpression and miR-484 inhibitor (Figures 5J,K). Knockdown of miR-484 partially reversed MAGI1 inhibition due to the silencing of TMEM220-AS1 in HuH-7 cells (Figure 5L). The results indicated that TMEM220-AS1 modulated MAGI1 expression in an miR-484-dependent manner in HCC cells. The transfection efficiency of miR-484 inhibitor and miR-484 mimics is shown in Supplementary Figures 1A,B.

### MAGI1 Inhibited the Proliferation, Invasion, and Tumor Formation of HCC

**FIGURE 6:**
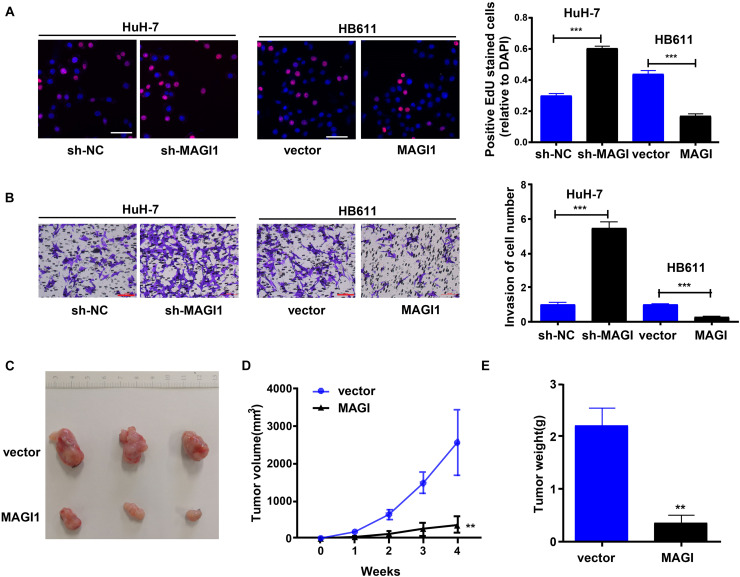
*MAGI1* inhibited the proliferation, invasion, and tumor formation of HCC. **(A)** Cell viability was analyzed by EdU assay (bar = 50 μm). **(B)** Transwell assays were used to determine the invasion of HCC cells (bar = 100 μm). **(C)** Representative image of tumors formed in nude mice from empty vector and *MAGI1* overexpression vector groups. **(D)** Tumor volume and **(E)** weight data of indicated orthotopic xenografts. **P < 0.01, ***P < 0.001.

To investigate the role of *MAGI1* in HCC, we used *MAGI1* shRNA to silence the expression of *MAGI1* in the HuH-7 cell line (Supplementary Figure 1C), and *MAGI1* overexpression vectors were used to increase the expression of *MAGI1* in the HB611 cell line (Supplementary Figure 1D). CCK-8 assays demonstrated that *MAGI1* knockdown inhibited HuH-7 cell growth, and *MAGI1* overexpression promoted HB611 cell growth (Figure 6A). Similarly, *MAGI1* knockdown inhibited the invasion of HuH-7 cells, and *MAGI1* overexpression promoted the invasion of HB611 cells (Figure 6B). *In vivo*, HB611 cells transfected with *MAGI1* overexpression vectors were subcutaneously injected into nude mice. The tumor lumps are shown in Figure 6C. The tumor volumes (Figure 6D) and weights (Figure 6E) in *MAGI1* overexpression group were clearly inhibited compared to those in the vector group.

### TMEM220-AS1/miR-484 Axis Regulates Behaviors of HCC Cells

**FIGURE 7:**
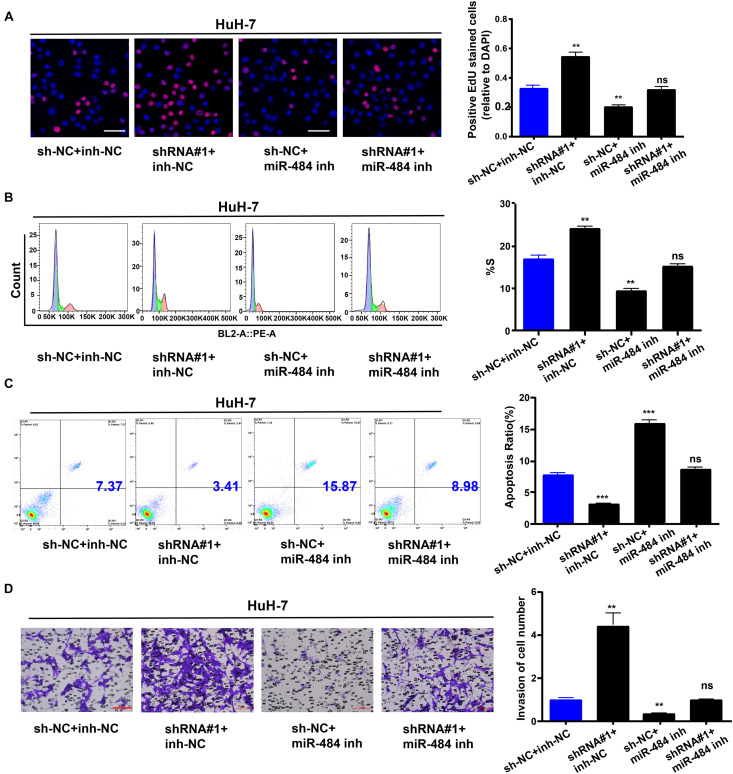
TMEM220-AS1/miR-484 axis regulates behaviors of HCC cells. **(A)** EdU assays were used to determine the cell proliferation ability of HuH-7 cells. **(B)** Cell cycle (bar = 50 μm). **(C)** Cell apoptosis. **(D)** Cell invasion (bar = 100 μm). Data were presented as represent the mean ± SD of 3 independent experiments; **P < 0.01, ***P < 0.001, ns, no significance.

Subsequently, we explored the effect of the TMEM220-AS1/miR-484 axis on HCC. We transfected HuH-7 cells and divided them into sh-NC + inh-NC, sh-TMEM220-AS1#1 (shRNA#1) + inh-NC group, sh-NC + miR-484 inh group, and sh-TMEM220-AS1#1 (shRNA#1) + miR-484 inh group. First, the EdU assay showed that cell proliferation was increased by silencing TMEM220-AS1, but it was decreased by miR-484 inhibitor, and miR-484 inhibitor treatment reversed the promoting effect of TMEM220-AS1 silencing on cell proliferation (Figure 7A). Next, the proportion of cells in the S-phase was increased by silencing TMEM220-AS1, while miR-484 inhibitor decreased the proportion of cells in S-phase. The effect of TMEM220-AS1 shRNA on the cell cycle was reversed by co-transfection with the miR-484 inhibitor (Figure 7B). In addition, the miR-484 inhibitor promoted cell apoptosis. Knockdown of TMEM220-AS1 downregulated cell apoptosis, but the effect of TMEM220-AS1 knockdown on cell apoptosis could be reversed by co-transfection with miR-484 inhibitor (Figure 7C). Finally, cell invasion was increased by silencing TMEM220-AS1; however, it was decreased by the miR-484 inhibitor. Moreover, miR-484 inhibitor treatment reversed the effect of TMEM220-AS1 silencing on cell invasion (Figure 7D).

### TMEM220-AS1 Overexpression Limited the Growth and Metastasis of HCC *in vivo*

**FIGURE 8:**
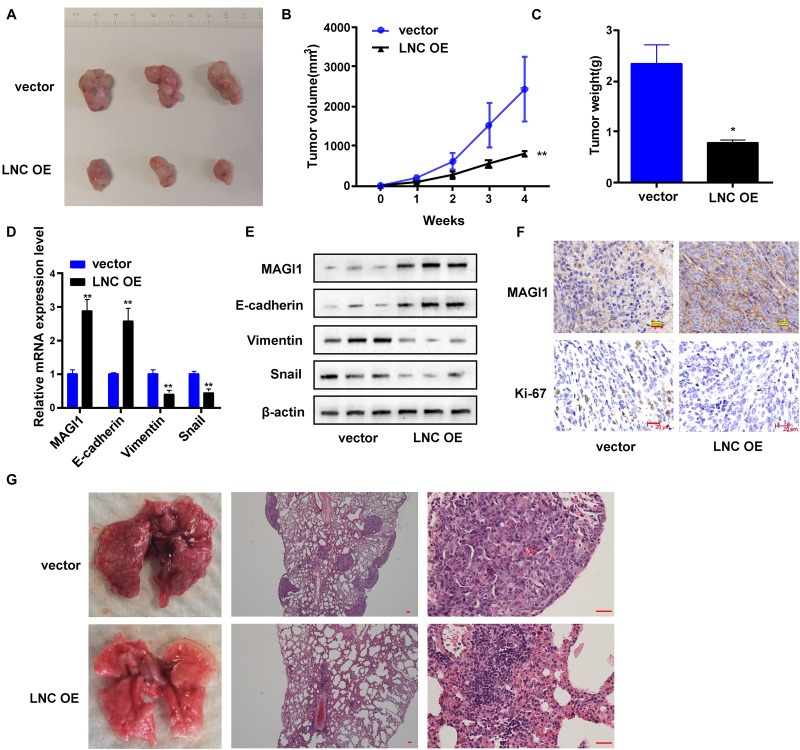
TMEM220-AS1 overexpression limited the growth and metastasis of HCC *in vivo.* **(A)** Representative images of tumors from indicated orthotopic xenografts. **(B)** Tumor volume and **(C)** weight data of indicated orthotopic xenografts. **(D,E)** mRNA and protein expression levels of MAGI1 and EMT-related markers after TMEM220-AS1 overexpression. **(F)** The expression level of MEGI1 and Ki-67 determined using immunohistochemistry (bar = 20 μm). **(G)** Representative images of lung metastases of indicated orthotopic xenografts (bar = 50 μm). Data were presented as represent the mean ± SD; *P < 0.05, **P < 0.01.

We generated xenograft models to verify the findings in this study. HB611 cells transfected with TMEM220-AS1 overexpression vectors were subcutaneously or intravenously injected into nude mice. The results showed that TMEM220-AS1 overexpression greatly limited tumor proliferation *in vivo* (Figures 8A–C). The tumors collected from the mice are shown in Figure 8A. Tumor growth in the vector group was faster than that in the TMEM220-AS1 overexpression group, both in volume and weight (Figures 8B,C). qRT-PCR and western blotting indicated that upregulation of TMEM220-AS1 increased the expression of MAGI1 and E-cadherin, but inhibited vimentin and Snail *in vivo* (Figures 8D,E). Immunohistochemistry also showed that TMEM220-AS1 promoted MAGI1 expression, but decreased Ki-67 expression in xenograft tumor tissues (Figure 8F). Moreover, TMEM220-AS1 overexpression in pulmonary metastasis models greatly decreased the incidence of pulmonary metastasis (Figure 8G). Thus, TMEM220-AS1 inhibits HCC growth and metastasis *in vivo*.

## Discussion

HCC is a frequently detected malignant tumor of the digestive system, and its occurrence is associated with the unrestricted proliferation of hepatocytes (Jin et al., 2018). Therefore, any cause of hepatocyte proliferation may lead to HCC. In recent years, lncRNAs have become a focus of tumor-related research, and there is much evidence that they can participate in the modulation of cancer cell migration, proliferation, and apoptosis (Chen et al., 2020; Nekvindova et al., 2020; Shang et al., 2020; Wang et al., 2020). In this study, TMEM220-AS1 was selected by analyzing TCGA database, which was poorly expressed in HCC samples and was associated with clinical staging and survival prognosis. Then, we verified the low expression of TMEM220-AS1 in a large population-based sample (*n* = 50), and the results of subsequent cell function experiments showed that the downregulation of TMEM220-AS1 promoted cell proliferation, cell cycle, invasion, and EMT process, while cell apoptosis was inhibited. Next, we studied the specific mechanism of TMEM220-AS1 in HCC.

It has been shown that lncRNAs can interact with miRNAs and regulate target mRNAs (Bo et al., 2020; Lyu et al., 2020). For example, AGAP2-AS1 promotes ANXA11 expression by sponging miR-16-5p and promotes proliferation and metastasis in HCC (Liu et al., 2019). Another study showed that the growth and epithelial-to-mesenchymal transition phenotype was regulated by the LINC01287/miR-298/STAT3 feedback loop in HCC cells (Mo et al., 2018). In addition, the migration and invasion of HCC cells were promoted by the lncRNA n335586/miR-924/CKMT1A axis (Fan et al., 2018). In our study, LncBase Experimental v.2 was used to predict miRNAs that might bind to TMEM220-AS1, and RNA pull-down, interference experiments with TMEM220-AS1, dual luciferase reporter assay, RIP, and qRT-PCR results indicated that TMEM220-AS1 acts as a molecular sponge for miR-484. Moreover, miR-484 has been reported to promote non-small cell lung cancer (Li et al., 2017) and HCC (Qiu et al., 2019) progression. Subsequent results also confirmed that miR-484 inhibitor curbed the invasion, proliferation, and cell cycle of HuH-7 cells and promoted the apoptosis of HuH-7 cells. Moreover, miR-484 inhibitor can partially reverse the effects of TMEM220-AS1 shRNA on the proliferation, invasion, cell cycle, and apoptosis of HCC cells.

The downstream target genes of miR-484 were predicted using MIRDB. The top five mRNAs (*MAGI1*, *TNRC6C*, *HOXA5*, *PTPRE*, and *ACVR1B*) according to their scores were selected as potential research subjects. Only *MAGI1* expression was inhibited by miR-484 overexpression in HCC cells. A dual luciferase reporter assay was performed to confirm the binding relationship between miR-484 and *MAGI1*. Some studies have indicated that in estrogen receptor-positive breast cancer, *MAGI1* is a new potential tumor suppressor gene (Alday-Parejo et al., 2020). Via the Wnt/β-Catenin and PTEN/AKT signaling pathways, *MAGI1* silencing inhibits apoptosis of glioma cells and promotes proliferation (Lu et al., 2019). Moreover, by regulating PTEN, *MAGI1* curbed the invasion and migration of HCC (Zhang and Wang, 2011). In summary, our study confirmed that *MAGI1* was the downstream target gene of miR-484, and TMEM220-AS1 released *MAGI1* through competitive binding of miR-484. *MAGI1* inhibited the proliferation, invasion, and tumor formation of HCC.

This research has several limitations. First, U6 and GAPDH (or 18S) should be added as controls in RNA-FISH assay. Second, it’s better to measure EMT-related proteins in lung metastasis by Immunochemistry or Immunofluorescence.

## Conclusion

In conclusion, TMEM220-AS1 acts as a tumor suppressor that inhibits HCC cell proliferation and metastasis, while promoting apoptosis through the miR-484/*MAGI1* axis.

## Data Availability Statement

The original contributions presented in the study are included in the article/Supplementary Material, further inquiries can be directed to the corresponding author/s.

## Ethics Statement

The studies involving human participants were reviewed and approved by the Affiliated Hospital of Youjiang Medical University for Nationalities. The patients/participants provided their written informed consent to participate in this study. The animal study was reviewed and approved by the Affiliated Hospital of Youjiang Medical University for Nationalities. Written informed consent was obtained from the individual(s) for the publication of any potentially identifiable images or data included in this article.

## Author Contributions

SC and JQL designed and supervised the study. CC, JL, and GL performed the experiments. GH and ZD collected and analyzed the data. BH and JY supported administration, technique and materials. CC prepared the manuscript. SC revised the manuscript. All authors read and approved the final manuscript.

## Conflict of Interest

The authors declare that the research was conducted in the absence of any commercial or financial relationships that could be construed as a potential conflict of interest.

## Publisher’s Note

All claims expressed in this article are solely those of the authors and do not necessarily represent those of their affiliated organizations, or those of the publisher, the editors and the reviewers. Any product that may be evaluated in this article, or claim that may be made by its manufacturer, is not guaranteed or endorsed by the publisher.
