# Supplementary material for: Long Non-coding RNA TMEM220-AS1 Suppressed Hepatocellular Carcinoma by Regulating the miR-484/MAGI1 Axis as a Competing Endogenous RNA
Source: Front Cell Dev Biol. 2021 Aug 5;9:681529. doi: 10.3389/fcell.2021.681529 (PMC8376477; doi:10.3389/fcell.2021.681529)
Supplement: Supplementary file 1 [file Data_Sheet_1.docx]

**Supplementary Figure 1**

**
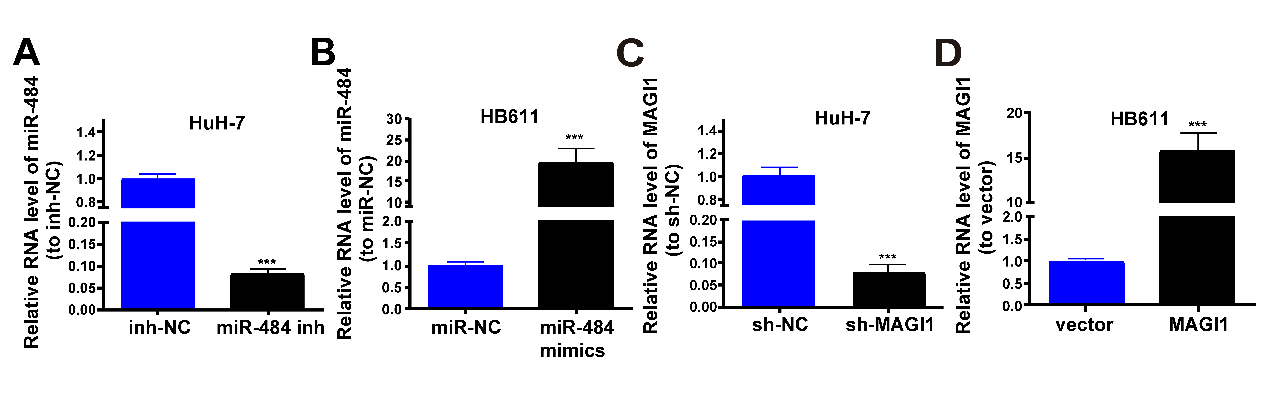
**

**Supplementary Figure 1. Transfection efficiency.** (A) Transfection efficiency of miR-484 inhibitor in HuH-7 cells. (B) Transfection efficiency of miR-484 mimics in HB611 cells. (C) Transfection efficiency of MAGI1 shRNA in HuH-7 cells. (D) Transfection efficiency of MAGI1 overexpression vectors in HB611 cells. Data represent the mean ± SD of 3 independent experiments; ***P < 0.001.
