# Supplementary material for: Long Non-coding RNA TMEM220-AS1 Suppressed Hepatocellular Carcinoma by Regulating the miR-484/MAGI1 Axis as a Competing Endogenous RNA
Source: Front Cell Dev Biol. 2021 Aug 5;9:681529. doi: 10.3389/fcell.2021.681529 (PMC8376477; doi:10.3389/fcell.2021.681529)
Supplement: Supplementary file 2 [file Data_Sheet_2.docx]

**Supplementary Methods**

1. Cell counting Kit-8 assay

Cell Counting Kit-8 (Beyotime Inst Biotech, China) was used to determine cell proliferation. In a word, we first cultured 5×10^3^ cells/well in a 96-well flat-bottomed plate at 37 °C for 1 day, followed by transfecting them with corresponding vectors. Finally, with a microplate reader (Bio-Rad, Hercules, CA, USA), the absorbance was finally evaluated at a wavelength of 450 nm. We carried out each experiment for three times.

2. 5-ethynyl-2’-deoxyuridine (EdU) incorporation assay

Cell proliferation was also determined by Ethynyl-2-deoxyuridine incorporation assay using an EdU Apollo DNA in vitro kit (RIBOBIO, Guangzhou, China) following the manufacturer’s instructions. Briefly, cells after transfection with the corresponding vector were respectively incubated for 2 h at 37 °C, with 100 μl of 50 μM EdU per well. Via a fluorescence microscopy, the cells were identified. We carried out each experiment for three times.

3. Cell cycle analysis

We utilized Cell Cycle and Apoptosis Kit (Beyotime, China) to detect cell cycle. Briefly, we washed cells with pre-cooled PBS and fixed them with 70% ethanol for 24 h. After that, 0.2% Triton X-100 was added to the cell solution, followed by re-suspension with 100 μg/mL RNase A for 30 min. Next, 10 μL PI solution was added for staining at 37 °C in the dark. BeamCyte (China) was used to analyze the results.

4. Measurement of apoptosis

Via Annexin V-FITC/PI Apoptosis Detection Kit (Beyotime, China), we detected apoptotic cells. Briefly, we seeded cells into a 6-well plate and treated them with different TiO2 NPs concentration for 24 h. Next, we washed the cells with PBS for three times and in the dark at room temperature stained them with 5 μL Annexin V-FITC for 5 min, followed by adding 10 μL Propidium Iodide (PI) and culturing for 15 min. The samples were detected with BeamCyte (China) and analyzed with CytoSY 1.0 software. Apoptotic cells were represented as the Annexin V (+) /PI (-) cells.

5. Transwell assay

Transwell chambers were used to observe the invasion of HCC cells. We seeded HCC cells in the upper chamber precoated with Matrigel (Corning, USA, dilution ratio: 1:6) at a density of 10^5^ cells per well and supplemented DMEM with 1% FBS. We filled 600μL DMEM with 10% FBS into the lower chamber. We fixed cells by 4% methanol and stained them with crystal violet. Then we counted them in 5 random 200X microscopic fields, after cells being invaded to the lower surface of membrane and incubated at 37°C for 24 hours. We carried out each experiment for three times.
